# Case Report of *Aerococcus urinae* Tricuspid Valve Endocarditis, New York, USA

**DOI:** 10.3201/eid3105.250013

**Published:** 2025-05

**Authors:** Tasfia Siam, Tyler Stephen, Sonal Munsiff

**Affiliations:** Shaheed Tajuddin Medical College, Gazipur, Bangladesh (T. Siam); University of Rochester Medical Center, Rochester, New York, USA (T. Stephen, S. Munsiff)

**Keywords:** *Aerococcus urinae*, endocarditis, heart valve prosthesis, tricuspid valve, case report, bacteria, New York, United States

## Abstract

We report a case of a 61-year-old man in New York, USA, who had recurrent *Aerococcus urinae* endocarditis that first involved his native and then his bioprosthetic tricuspid valve. We demonstrate that a complicated *A. urinae* endocarditis case can be successfully treated with single-agent antimicrobial drug therapy and surgery.

*Aerococcus urinae* is a gram-positive, catalase-negative, α-hemolytic coccal bacterium predominantly found in the urinary tract. Historically, *A. urinae* has been frequently misidentified because of limitations in biochemical testing. However, advances in microbiology have enabled more reliable identification (*1*). Infective endocarditis caused by *A. urinae* is rarely reported (*2*). We report a case of *A. urinae* endocarditis in a patient who had a previous bioprosthetic valve replacement because of endocarditis caused by this organism. Written consent was obtained from the patient for publication of clinical information and photographs.

A 61-year-old man sought care at an emergency department in New York, USA, after a fall at home. Three weeks before seeking care, he had stopped taking torsemide, metformin, and lisinopril at his home and had switched to a carnivore diet consisting of only meat. Two weeks before seeking care, he experienced recurrent falls, progressive general weakness, fatigue, dyspnea on exertion, and decreased oral intake; painful hand and foot lesions also developed. He denied having fevers, chills, chest pain, or urinary symptoms before hospital admission. His medical history included chronic kidney disease, bulbar urethral stricture with prior perineal urethrostomy 8 years before, type 2 diabetes mellitus, and coronary artery disease. In addition, he had been admitted 14 months before for native tricuspid valve endocarditis caused by *A. urinae* and for L2–L3 lumbar discitis. He was treated for 6 weeks with intravenous ceftriaxone for endocarditis and osteomyelitis. Two weeks into therapy, he underwent bioprosthetic tricuspid valve replacement and coronary artery bypass graft surgery. Pathologic examination indicated acute endocarditis with fibrinosuppurative vegetations; however, Gram stain results were negative, and no organisms were isolated.

At emergency department admission, the patient was afebrile. Physical examination revealed a new III/VI systolic murmur and reddish/purple papular, nonblanching rashes on all 4 distal extremities (Figure 1). The patient’s serum creatinine level was 4.13 mg/dL, glomerular filtration rate was 16 mL/min/1.73 m^2^, and blood hemoglobin A1C was 10.0%. Urinalysis showed 3+ blood, 2+ leukocyte esterase, 6–10 leukocytes/high-power field, and no bacteria. No sample was sent for culture because of minimal pyuria. Blood cultures grew *A. urinae* (penicillin MIC <0.03 µg/mL, ceftriaxone MIC <0.12 µg/mL). A transthoracic echocardiogram showed large masses involving all bioprosthetic tricuspid valve leaflets, leading to reduced excursion and severe valve stenosis (Figure 2). Magnetic resonance imaging of the lumbar spine showed no evidence of active osteomyelitis or discitis. Bladder examination showed postvoid residual volumes within reference ranges, and prostate ultrasound results were unremarkable. Biopsies of skin lesions indicated leukocytoclastic vasculitis.

**Figure 1:**
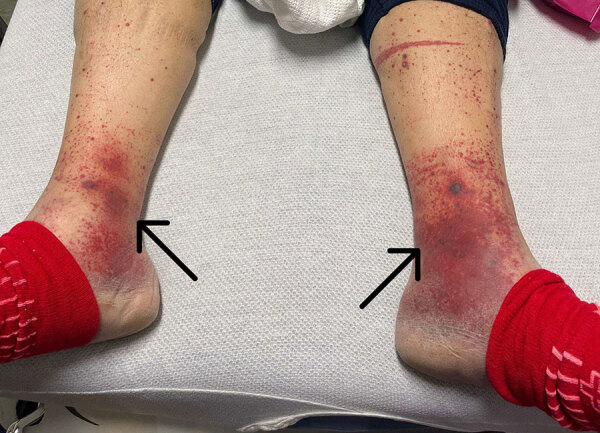
Extremity rash on patient from case report of *Aerococcus urinae* tricuspid valve endocarditis. Rash (arrows) was documented at the time the patient sought care.

**Figure 2:**
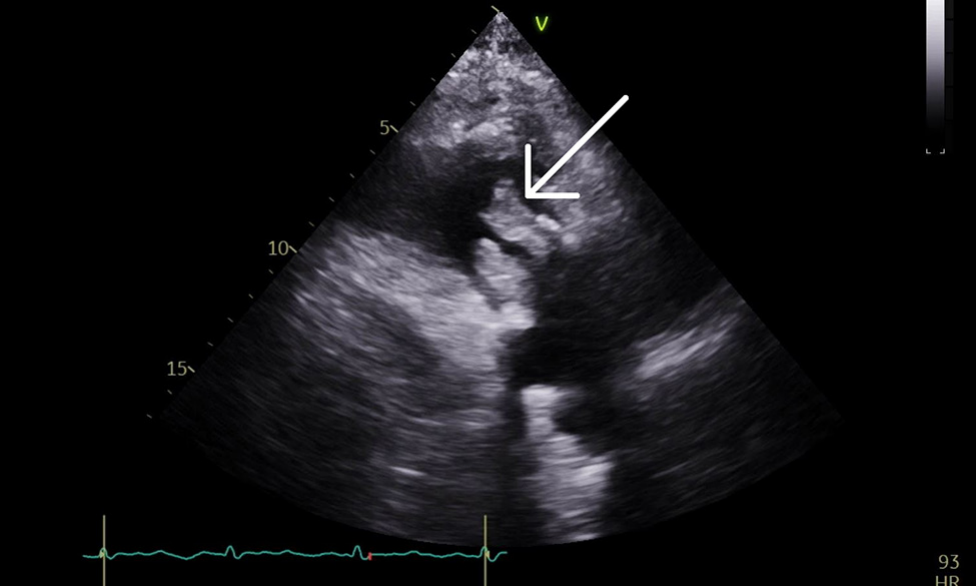
Echocardiogram of patient from case report of *Aerococcus urinae* tricuspid valve endocarditis. Arrow indicates tricuspid mass. V at top of image indicates positive inflection. Numbers on the left side indicate depth from the ultrasound probe (in cm). Electrical signal profile is indicated at the bottom of the image.

We diagnosed infective endocarditis associated with vasculitis and immune complex–mediated glomerulonephritis. We considered the infection to be recurrent rather than relapsed. He underwent intravenous ceftriaxone (2 g/d) treatment while awaiting drug susceptibility testing, and his blood cultures cleared within 24 hours. After MIC testing indicated antimicrobial drug susceptibility, we switched his treatment to renally dosed, continuous infusion penicillin G (12 million units/d). Because of large vegetations and valvular dysfunction, he underwent a repeat tricuspid valve replacement 4 weeks into therapy; tissue cultures were negative, and pathologic examination showed inflammatory cells and fibrin without organisms. He completed 6 weeks of parenteral antimicrobial drug therapy without adverse drug effects. Nine months after completion of antimicrobial drug therapy, he had no residual or recurrent symptoms.

*A. urinae* and other aerococci are more common as uropathogens. Although the urinary tract is believed to be the initial source of bacteremia and endocarditis in many reports, aerococcal infections might remain unidentified in urinary cultures because of the lack of optimal (5%) carbon dioxide exposure during laboratory incubation (*2*,*3*). Risk factors for aerococcal endocarditis include older age, male sex, urinary tract infection, prostate hyperplasia, urethral strictures, urinary tract surgery, and urinary catheter usage (*4*,*5*). Our patient’s previous urethral stricture had been addressed by perineal urethrostomy years before this case, and he was functioning without urinary symptoms, although that history appears to be his strongest predisposing factor. It is unclear whether his dietary change and discontinuation of cardiac and diabetes medications directly contributed to his more recent illness.

A review of 58 cases of *Aerococcus* endocarditis revealed that mitral and aortic valve involvement was predominant; only 2 cases implicated the tricuspid valve, and only 2 cases involved prosthetic valves (*6*). Additional cases showed endocarditis affected a native tricuspid valve and a prosthetic aortic valve (*7*,*8*). This case showed a less commonly reported prosthetic tricuspid valve endocarditis.

Case-fatality rates of 14%–27% have been reported for *Aerococcus* endocarditis in case series, underscoring an opportunity and need for improved care (*7*,*9*). Clinical studies to determine optimal doses and duration of therapy against aerococcal infections have not been performed. *Aerococcus* spp. consistently show susceptibility to vancomycin, but some species show decreased susceptibility to penicillin and sulfamethoxazole. In vitro bactericidal synergism with β-lactams and daptomycin or an aminoglycoside has also been reported (*2*,*10*). Surgical intervention is not always required because many cases respond well to antimicrobial drug therapies (*1*). However, this case demonstrates successful treatment of a complicated case by using single-agent antimicrobial drug therapy and surgery.
